# Supplementary material for: The immune synapses reveal aberrant functions of CD8 T cells during chronic HIV infection
Source: Nat Commun. 2022 Oct 28;13:6436. doi: 10.1038/s41467-022-34157-0 (PMC9616955; doi:10.1038/s41467-022-34157-0)
Supplement: Supplementary file 1 — Supplementary Information [file 41467_2022_34157_MOESM1_ESM.pdf]

SUPPLEMENTARY INFORMATION

Supplementary Table 1. Clinical characteristics of HIV-infected donors*)

	HIV+	HIV+ ART
Number of individuals	8	5
CD4 count (cells/ml)**)	382<br>(320-480)	550<br>(396-720)
Viral load (copies/ml)**)	43,789<br>(26,566-43,789)	<50

*)The interval of donors’ age in each subgroup was relatively narrow (range 19-39 years).
**) Median and interquartile range

Supplementary Table 2. **Antibodies used in this study**

Antibody	Source/ Catalog #	Clone	Fluorophore	Lot #	Dilution	Purpose
Anti- CD45RO	BD Biosciences #562299	UCHL1	PE-CF594	8067791	1:50	Flow sorting
Anti-CD56	BD Pharmingen #560916	B159	PE-Cy7	7319530	1:100	Flow sorting
Anti-CD45RA	BD Biosciences #563963	HI100	BV650	8129909	1:166.7	Flow sorting
Anti-CCR7	Biolegend #353211	G043H7	APC-Cy7	B314830	1:50	Flow sorting
Anti-CD27	Biolegend #302827	O323	BV785	B264783	1:66.7	Flow sorting
Anti-CD10	Biolegend #312222	HI10A	BV605	B217326	1:100	Flow sorting
Anti-CD14	Biolegend #301842	M5E2	BV510	B251706	1:200	Flow sorting
Anti-CD19	Biolegend #302208	HIB19	PE	B273506	1:200	Flow sorting
Anti-CD16	Biolegend #302048	3G8	BV510	B202740	1:200	Flow sorting
Anti-CD4	Biolegend #300512	RPA-T4	PE-Cy7	B240351	1:333.3	Flow sorting
Anti-CD3	ATCC CRL-8001	OKT3	Biotin and Alexa 488	In-house produced	2 μg/ml	Imaging
Anti-CD54	ATCC CRL-1878	YN1/1	Unlabeled	In-house produced	Immo-bilized	ICAM-1 purificatio n
Anti-CD11a	ATCC HB-244	TS2/4.1.1	Alexa 488	In-house produced	2 μg/ml	Flow cytometry
Anti-CD107a	DSHB H4A3	H4A3	Alexa 568	In-house produced	2 μg/ml	Imaging
Anti-CD8	BD Horizon #561617	SK1	V500	3200606	1:200	Flow Cytometry
Anti-CD27	Biolegend #302812	O323	Alexa 647	235714	1:100	Flow Cytometry
Anti-CD45RO	Biolegend #304232	UCHL1	BV650	B256838	1:100	Flow Cytometry

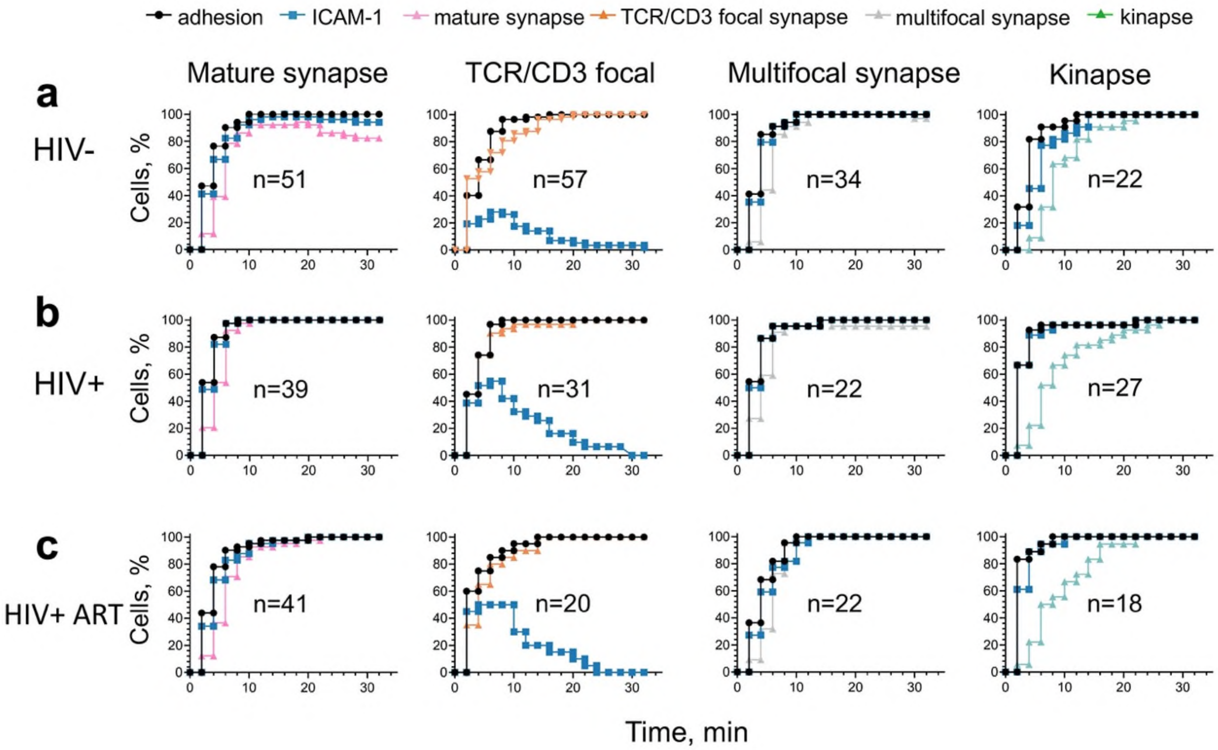

Supplementary Figure 1. Dynamics of CD8 T cells interaction with bilayer surface. CD8 T cells were isolated from uninfected donors by negative magnetic sorting and were exposed to bilayers presenting ICAM-1 and anti-CD3 antibody molecules. The images were acquired by confocal microscope every 2 min for 30 min. The cells adherence was tracked over time, and percentages of adhered cells were determined. Each adhered cell was assigned to one of four categories based on the interface type (see Figure 1). The interface type indicated on the top of the graphs. Percentages of adherent cells that accumulated ICAM-1 at the T cell/bilayer interface were determined for each time point and cell category. The data are presented as time-dependent graphs for cells from uninfected individuals (**a**), HIV-infected donors (**b**), and HIV positive individuals under ART (**c**). The interface type indicated on the top of the graphs; numbers of the analyzed cells indicated for each graph. One independent experiment is shown for each donor group; HIV-: N=3, HIV+: N=2, and HIV+ ART: N=2. Source data are provided as a Source data file.

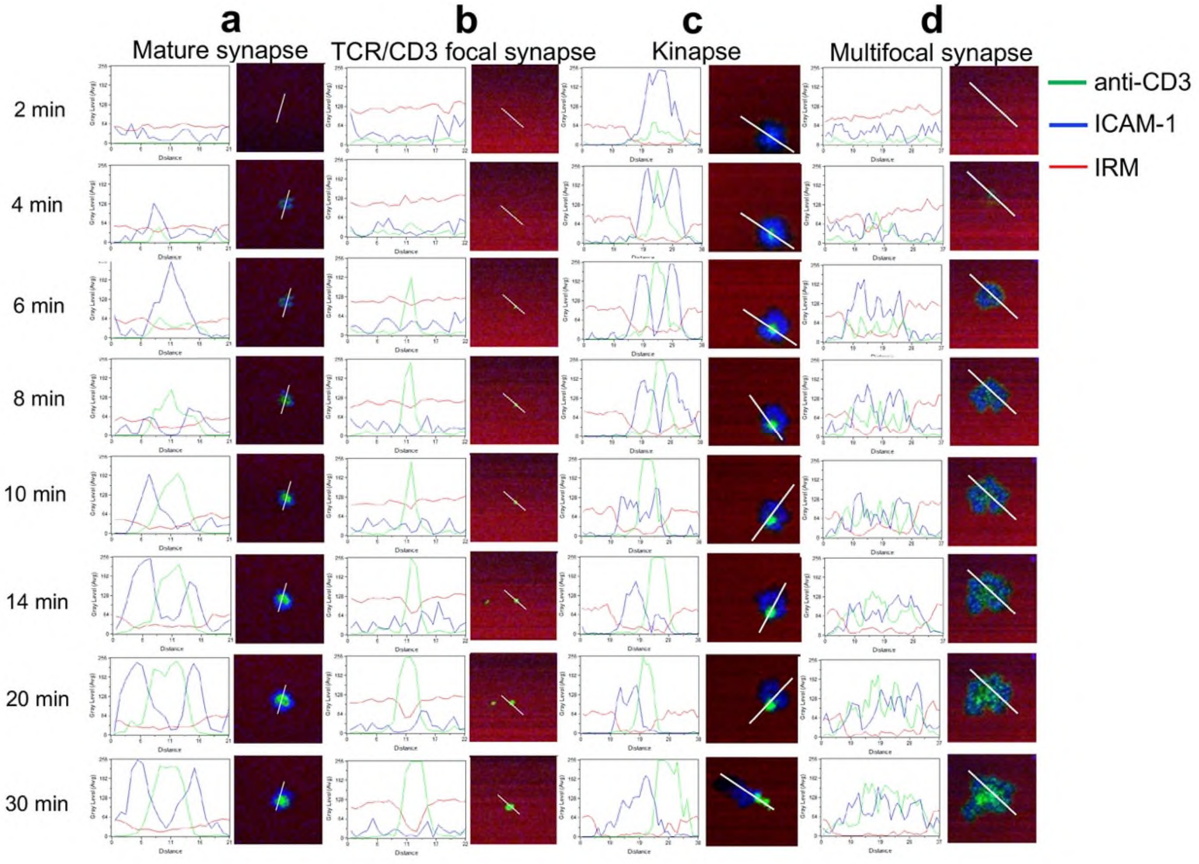

Supplementary Figure 2. Dynamics of the interface between live T cells and lipid bilayers during the process of the synapse formation. Isolated CD8 T cells were placed on ICAM-1 and anti-CD3 reconstituted lipid bilayers and imaged during 30 minutes at 2 min intervals with confocal microscopy. Representative overlay images and histograms show changes in the interface over time for cells that form mature synapse (**a**), TCR/CD3 focal synapse (**b**), kinapse (**c**) and multifocal synapse (**d**). Histograms depict the intensity profiles on the white lines in corresponding overlay images. ICAM-1 accumulation is in blue, anti-CD3 antibody accumulation is in green, IRM signal that reflect adhesion of cells to the bilayer surface is in red.

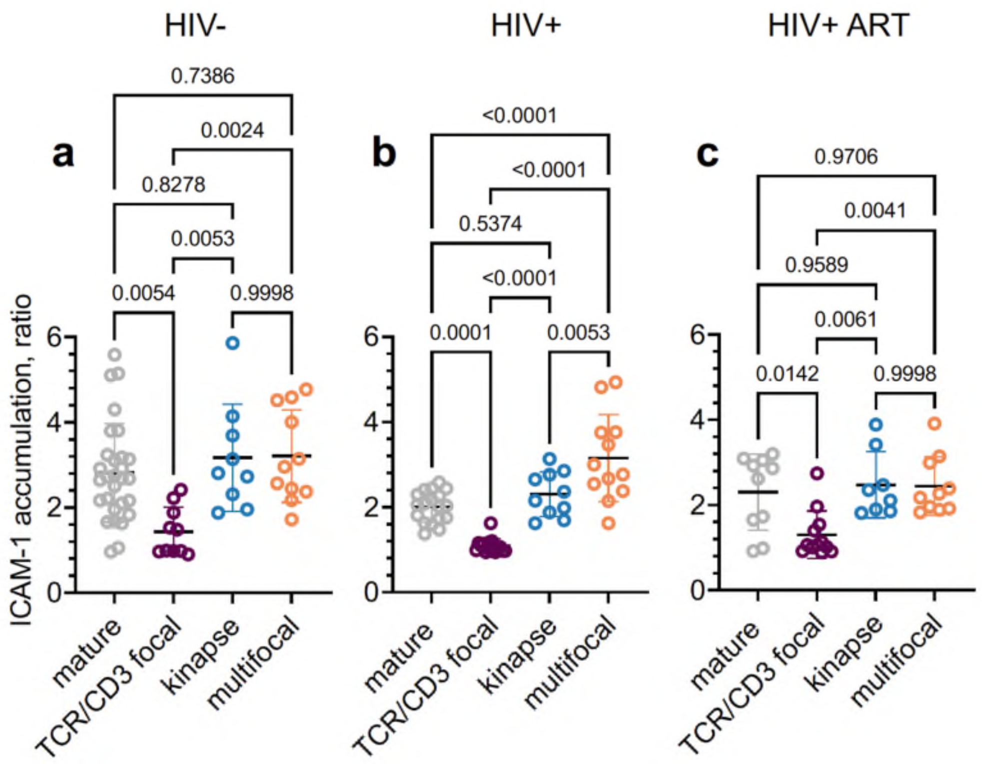

Supplementary Figure 3. ICAM-1 accumulation is similar at all kinds of synaptic interfaces except for TCR/CD3 focal one. CD8 T cells were purified from PBMC of HIV- (**a**), chronic HIV+ (**b**) and ART-treated donors (**c**) by negative magnetic sorting and were loaded onto bilayer surface presenting fluorescent-labelled anti-CD3 antibodies and ICAM-1 molecules. The contact interfaces were imaged by confocal microscopy, and the extent of ICAM-1 accumulation was calculated as a ratio of fluorescent intensity of the ICAM-1 enriched T cell contact area over ICAM-1 fluorescent intensity of a region adjacent to the contact area. The fold difference was examined for the cells establishing mature synapse (red empty circles), TCR/CD3 focal interfaces (black empty circles), kinapses (blue empty circles) and multifocal synapses (green empty circles) at 10 minutes after the T cell loading. Means with SD are indicated by black lines and error bars. The dots on the graphs present individual cells forming mature, TCR/CD3 focal, kinapse and multifocal synapse (HIV-: n=29, 10, 9, 11; HIV+: n=18, 16, 10, 12; HIV+ART: n=10, 12, 8, 10 cells, respectively). Means with SDs are indicated**.** Adjusted p values on a top of the graphs were calculated by ordinary one-way ANOVA with Tukey multiply comparison test. Representative results of one independent experiment for each donor group are shown; HIV-: N=3; HIV+: N=2, and HIV+ ART: N=2. Source data are provided as a Source data file.

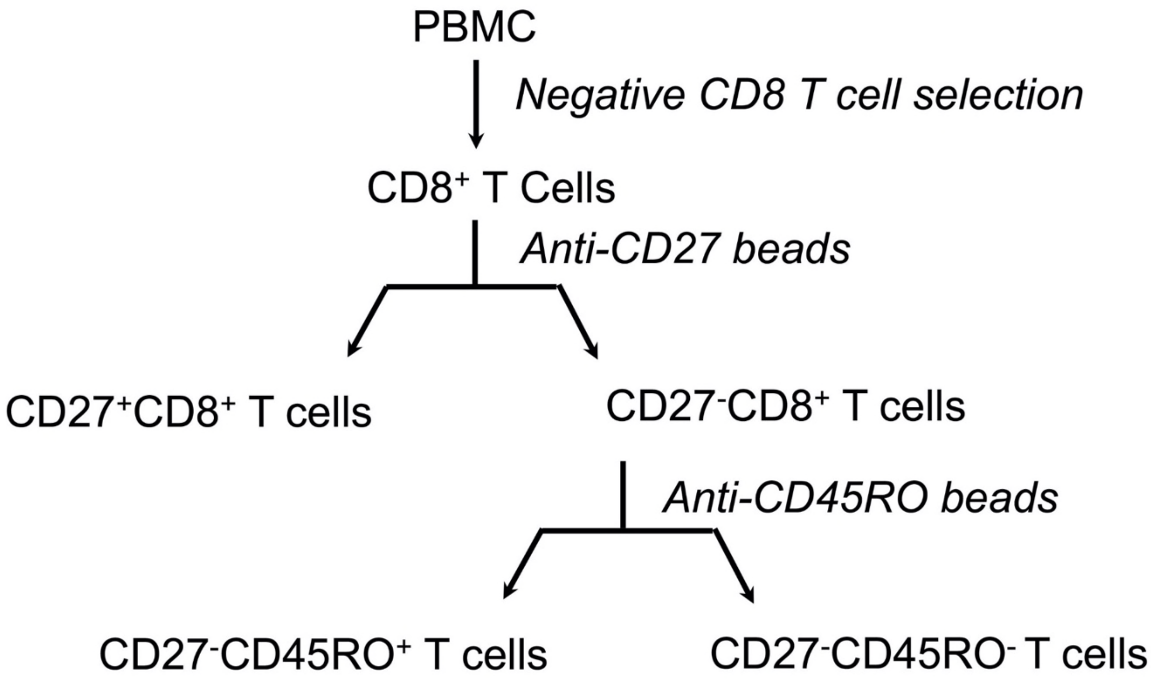

Supplementary Figure 4. Isolation of CD8 T cells at different stages of differentiation using magnetic sorting. CD8 T cells were isolated from PBMC by negative magnetic sorting. The cells were labelled with anti-CD27 magnetic microbeads and were positively selected using MACS separating column. The eluted fraction represents early differentiated CD27+ CD8 T cells. The CD27- depleted fraction were further labeled with anti-CD45 magnetic microbeads. The second round of separation generated CD27-CD45RO+ CD8 T cell subset and CD27-CD45RO- CD8 T cells at late differentiation stage.

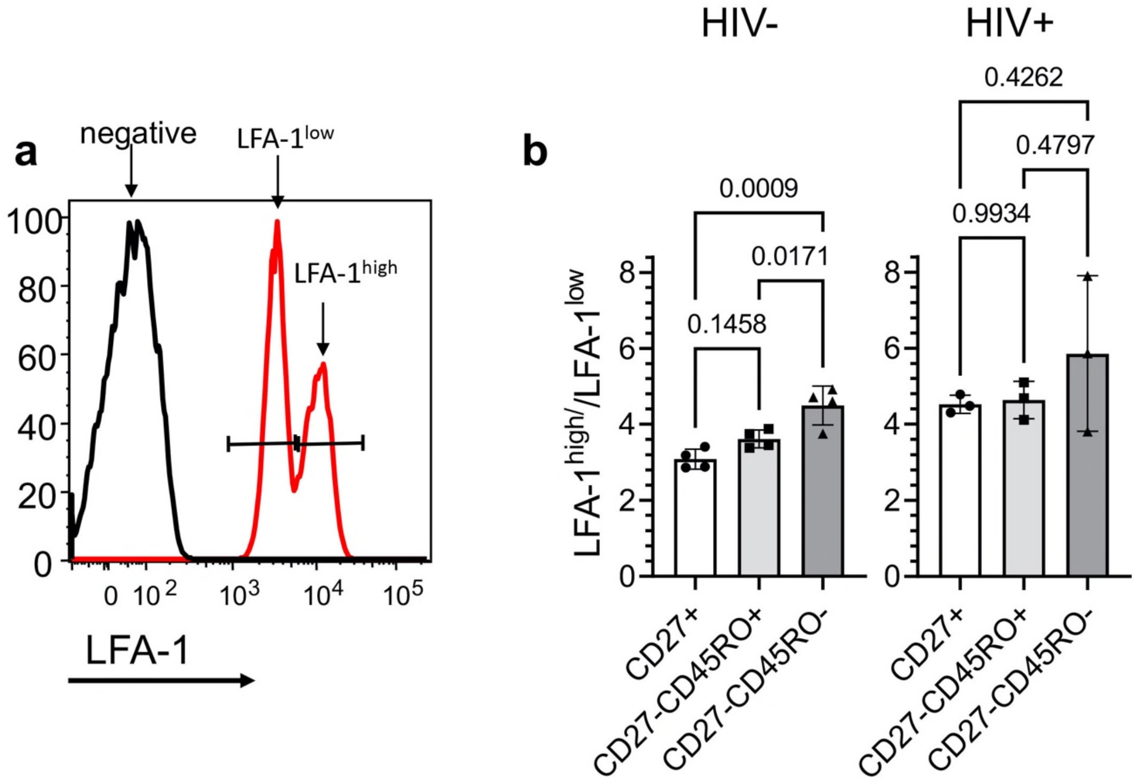

Supplementary Figure 5. Gating strategy for analysis of T cell fractions with distinct expression level of LFA-1. CD27+, CD27-CD45RO+ and CD27-CD45RO- CD8 T cell subsets were purified from PBMC by magnetic sorting. The cells were stained with 2 μg/ml Alexa Fluor 488 labelled non-stimulatory antibodies against LFA-1 (clone TS2/4) and analyzed by Flow Cytometry. Fluorescent intensity of Alexa Fluor 488 calibration beads (Bangs laboratory) was recorded at the same day and instrument settings for quantifying numbers of LFA-1 molecules per cell.

(**a**) Representative histogram shows gating strategy for LFA-1^{low} and LFA-1^{high} fractions of CD27+ CD8 T cell subset isolated from HIV- individual. (**b**) Ratio LFA-1^{high}/LFA-1^{low} molecules per cell for CD8 T cells at various differentiation stages derived from HIV-, HIV+, and ART-treated HIV+ people. The same gates shown in panel (**a**) were used for the analysis of all T cell subsets. Mean and SD is indicated for each T-cell subset. The differences between T-cell subsets within a subject group were analyzed by ordinary one-way ANOVA with Tukey multiply comparison test. HIV-: N=4, and HIV+: N=3 and HIV+ ART: N=3 independent experiments. Source data are provided as a Source data file.

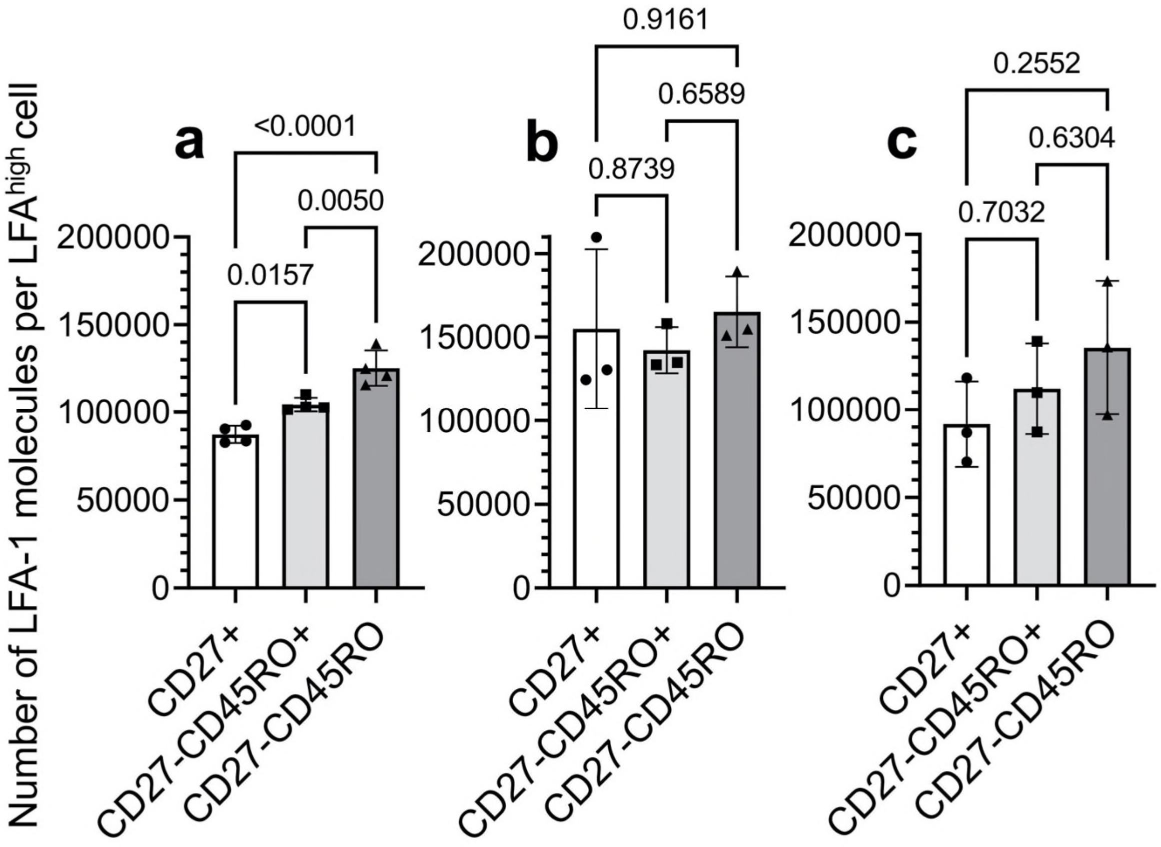

Supplementary Figure 6. Quantitation of LFA-1 expression level on CD8 T cell subsets. CD27+, CD27-CD45RO+, and CD27-CD45RO- CD8 T cell subsets from HIV- (**a**), HIV+(**b**), and ART-treated HIV+ (**c**) individuals were analyzed. The T cells were stained with either anti-LFA-1 (clone TS2/4) or isotype matched antibodies labeled with Alexa Fluor 488. Fluorescent intensity of Alexa Fluor 488 calibration beads (Bangs laboratory) were measured at the same instrument settings to quantify the number of LFA-1 molecules per cell. For each condition, mean with SD shown as bar graph with error bar. Each dot point represent independent experiment; HIV-: N=4, HIV+: N=3, HIV+ART: N=3. Exact p values calculated by ordinary one-way ANOVA with Tukey multiply comparison test and indicated on the top of the graphs. Source data are provided as a source data file.

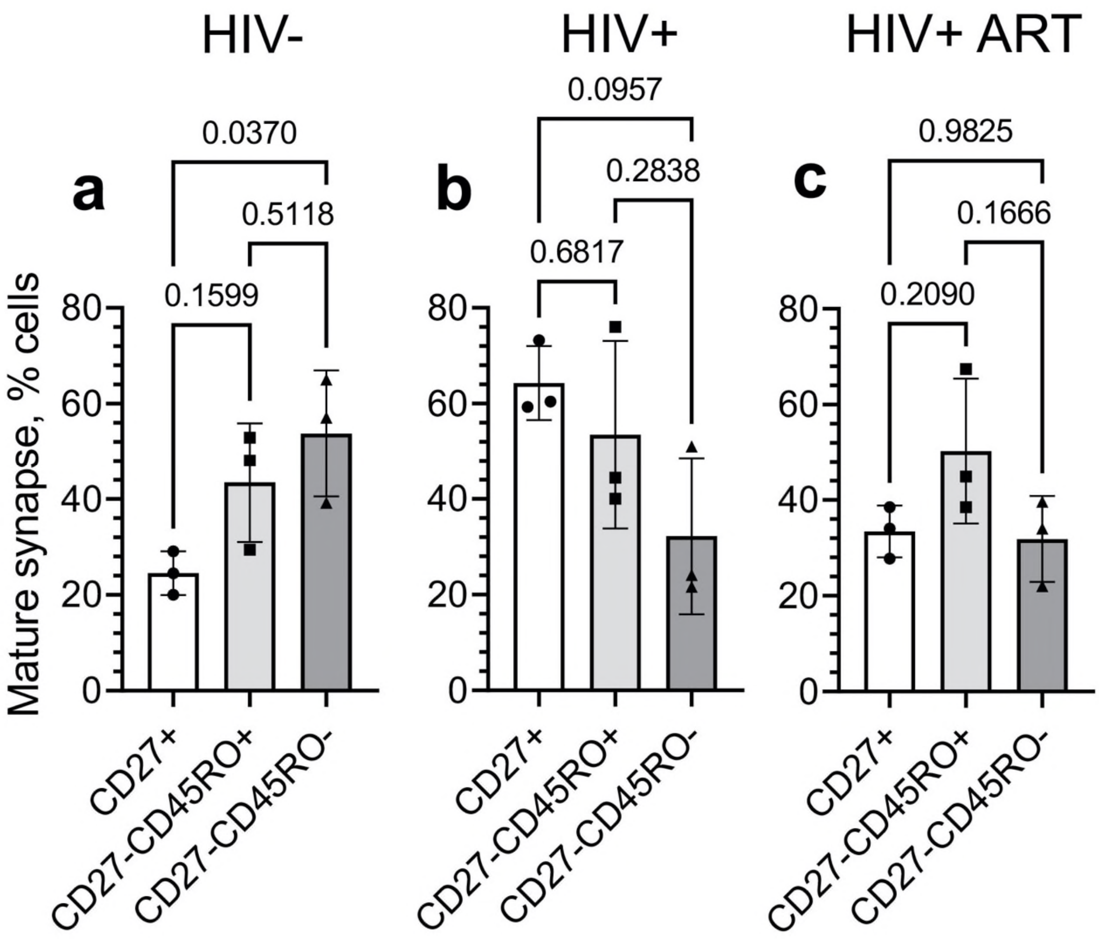

Supplementary Figure 7. Ability to form mature synapse during differentiation have upward trend only for CD8 T cells from HIV- donors. CD8 T cell subsets from HIV- (**a**), HIV+(**b**), and ART-treated HIV+ (**c**) individuals were exposed to bilayer surfaces presenting anti-CD3 antibodies and ICAM-1 molecules. Serial images of T cell/bilayer interface were taken for 30 min by confocal microscopy, and number of the cells establishing mature synapses were determined. The bar graphs show means with SDs. Comparison between T cell subsets within a subject group was performed by ordinary unpaired one-way ANOVA with Tukey multiply comparison test; exact p values are indicated. Each dot represent independent experiment; N=3 independent experiments for each group of donors with different infection status. Source data are provided as a source data file.

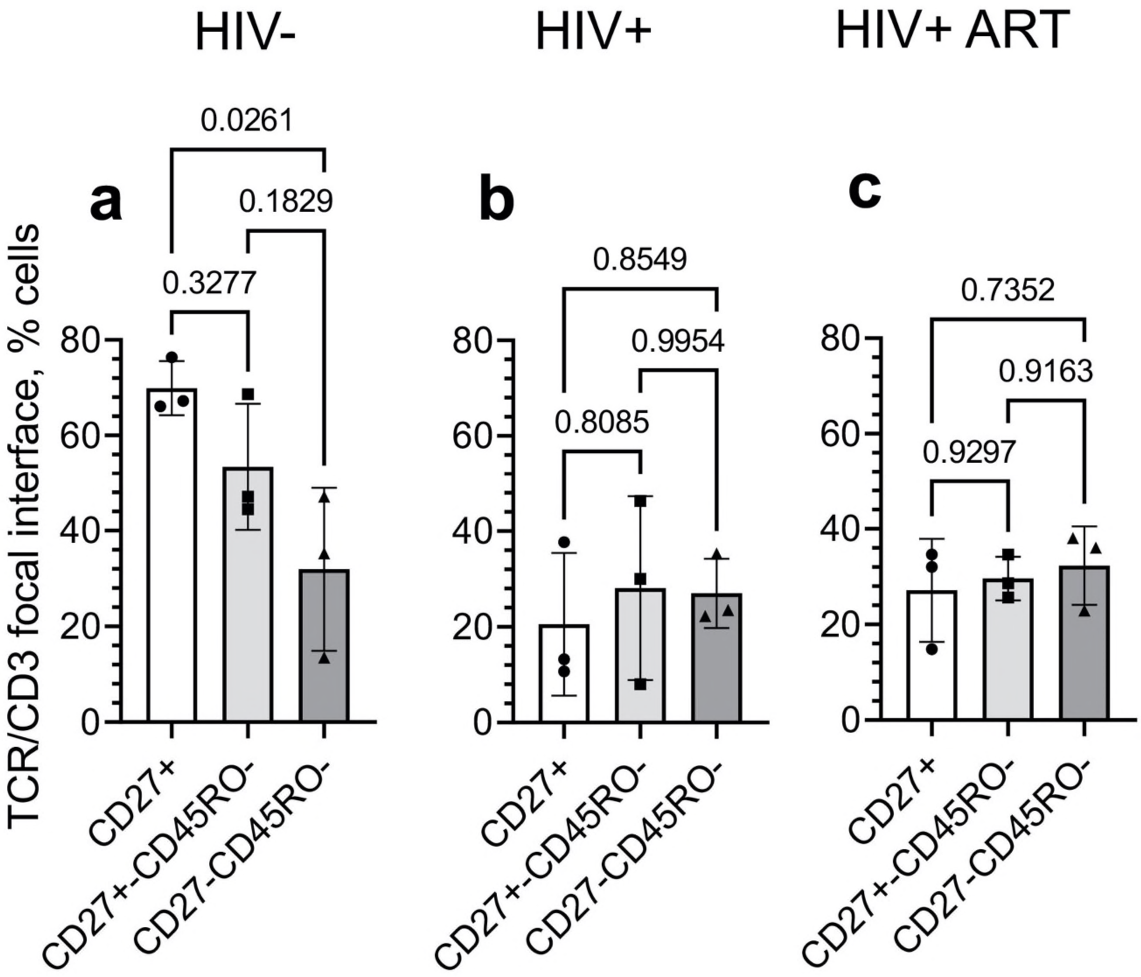

Supplementary Figure 8. In contrast to CD8 T cells from HIV- donors, the T cells from ART-treated and untreated HIV+ individuals revealed small fractions of T cells with CD3/TCR focal interfaces regardless of differentiation stage. CD8 T cell subsets from HIV- (**a**), HIV+(**b**), and ART-treated HIV+ (**c**) individuals were exposed to bilayer surfaces presenting fluorescent-labeled anti-CD3 antibodies and ICAM-1 molecules. The formation of synaptic interfaces was observed for 30 minutes by confocal microscopy at rate 2 frame/min. Number of the cells capable to form CD3/TCR focal interface were determined. For each donor group, means with SDs shown as bar graphs with error bars. Each dot point represents independent experiment; N=3 for each donor group. Exact p values calculated by ordinary one-way ANOVA with Tukey multiply comparison test and indicated on the top of the graphs. Source data are provided as a source data file.

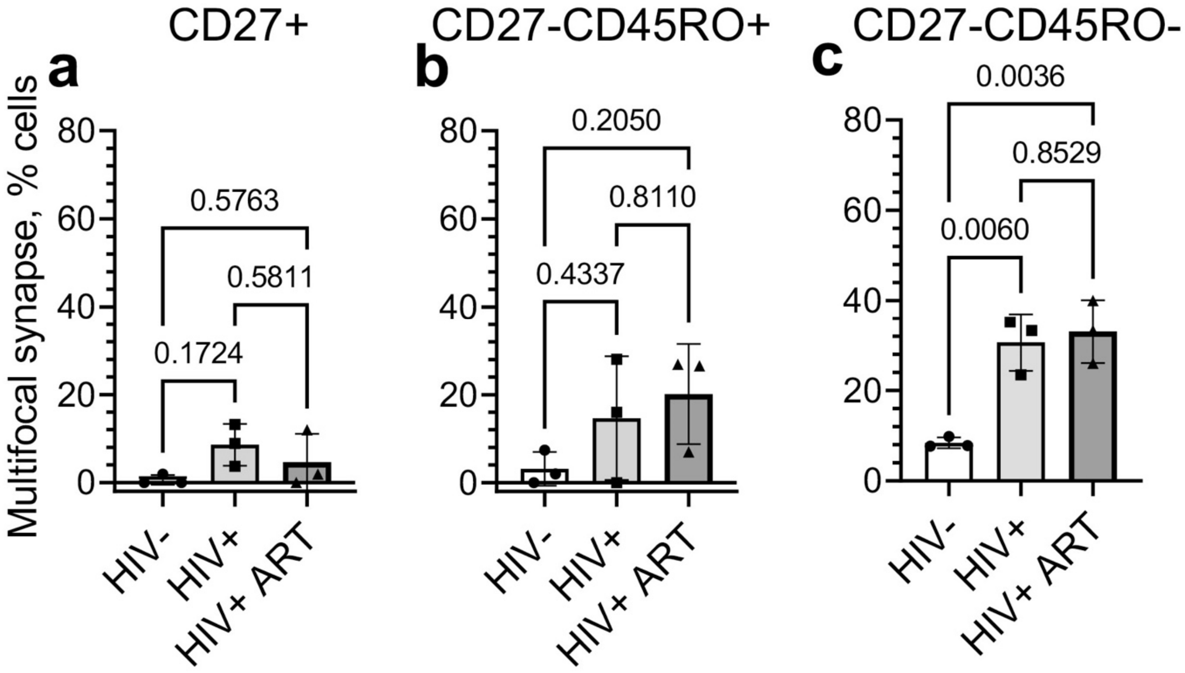

Supplementary Figure 9. CD8 T cells from HIV+ individuals capable to form noteworthy number of multifocal synapses at late differentiation stage. CD8 T cell subsets from HIV- (**a**), HIV+(**b**), and ART-treated HIV+ (**c**) individuals were loaded onto bilayer surfaces containing fluorescent labeled anti-CD3 antibodies and ICAM-1 molecules. Interfaces formed between bilayers and T cells were observed with confocal microscopy for 30 min. The bar graphs represent mean values (±SD) of three independent experiment for each donor group with different infection status**.** Statistical significance between subject groups was determined using one-way ANOVA with Tukey multiple comparison test, exact p values indicated on the graphs. Source data are provided as a source data file.

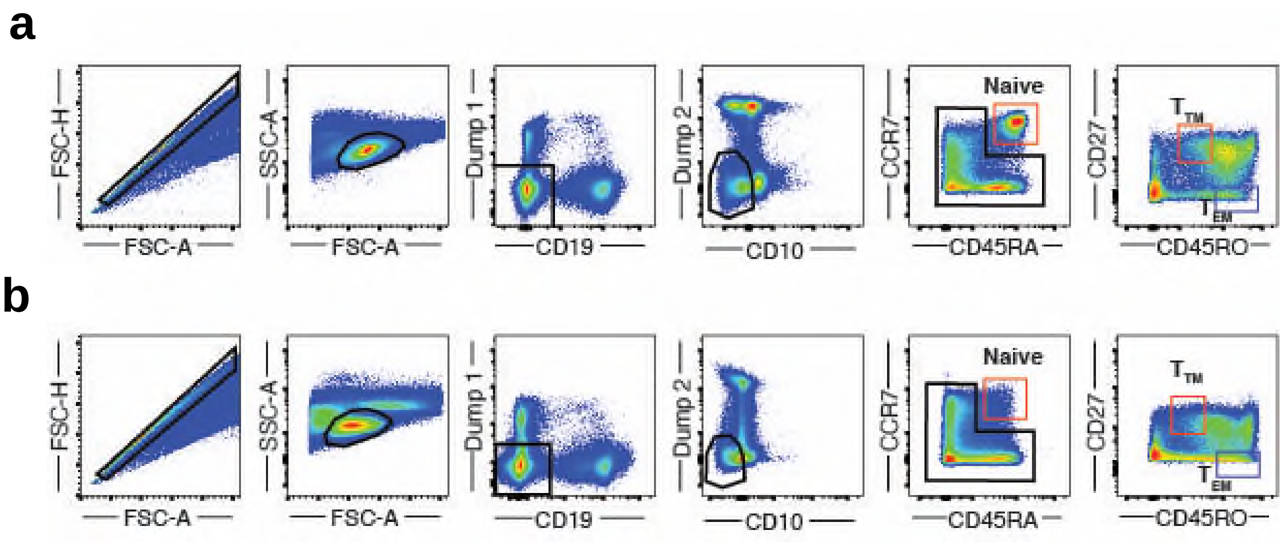

Supplementary Figure 10. Gating strategy for flow cytometry sorting of CD8+ T cell subsets.

Single lymphocytes were first characterized by morphology. Dead cells, CD14+ and CD16+ cells were excluded using Dump 1 (anti-Human CD14, anti-Human CD16, LIVE/DEAD™ Fixable Aqua Dead Cell Stain Kit, see Methods), and CD19- cells were gated. CD4+ and CD56+ cells were then excluded using Dump 2 (anti-Human CD56, anti-Human CD4, see Methods), and CD10+ cells were gated out. Naïve cells were characterized as CD45RA+ CCR7+ cells. Transitional (T_{TM}, red gate) and effector (T_{EM}, blue gate) were identified within the non-naïve cells. T_{TM} were characterized as CD27+ CD45ROlow, and T_{EM} were identified as CD27- CD45RO+ cells. **a**) Representative example on an HIV-negative individual. **b**) Representative example on an HIV-positive viremic individual. In HIV infected donors, the mean of CD4 count (±SD) was 493±76 cells/µl, and mean of HIV viral load (±SD) was 44,127±25,280 copies/ml.

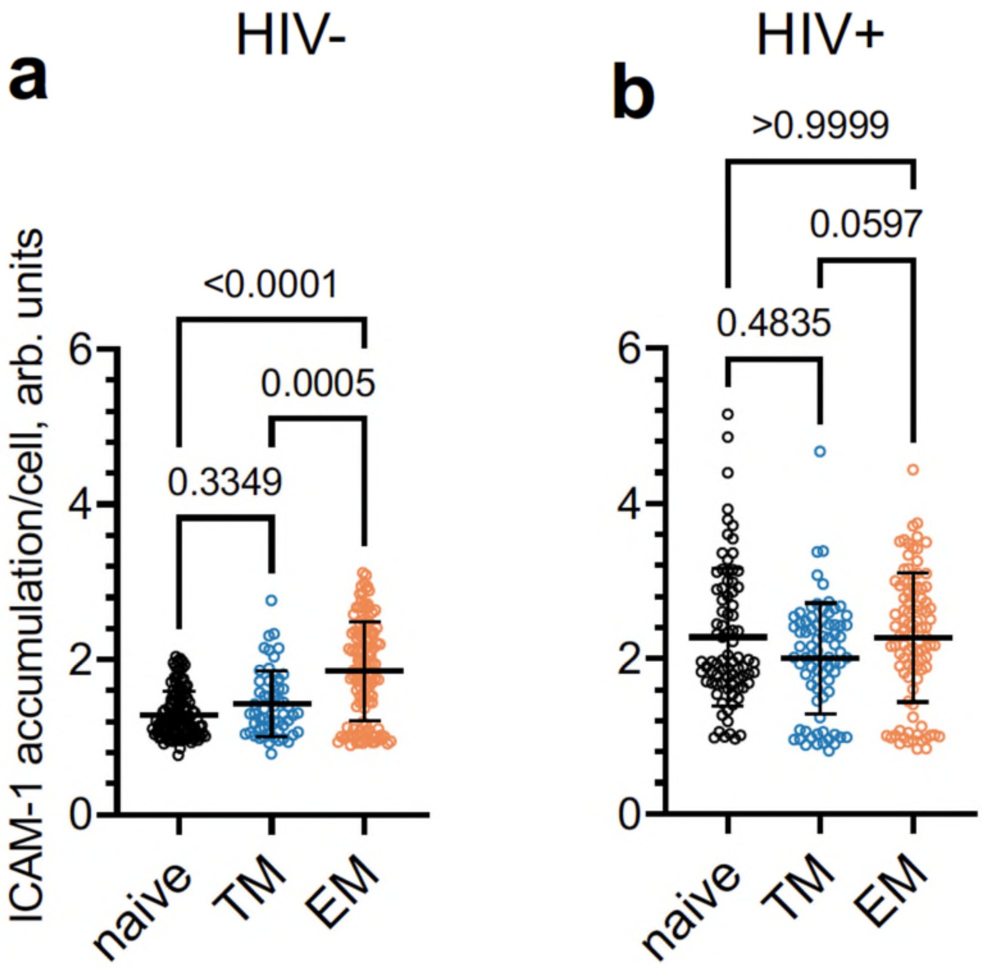

Supplementary Figure 11. **Extent of ICAM-1 accumulation at T cell/bilayer interface for CD8 T cell subsets (naïve, TM and EM) isolated from uninfected (a) and HIV-infected (b) individuals**. The extent of ICAM-1 accumulation was measured for adhered T cells as ratio of the average fluorescence intensity of accumulated Cy5-labeled ICAM-1 molecules at the cell-bilayer interface to background fluorescence outside of the contact areas. Each dot represents individual CD8 T cell; HIV-: n=121 (naïve), 57 (TM),124 (EM) and HIV+: n=82 (naïve), 78 (TM), 98 (EM) cells. Representative experiment is shown, N=2 (HIV- group) and N=3 (HIV+ group). For each kind of T cell subset, bar with error bars represent mean with SD. Exact p values were determined by nonparametric Kruskal-Wallis test with Dunn's multiply comparison. Source data are provided as a source data file.

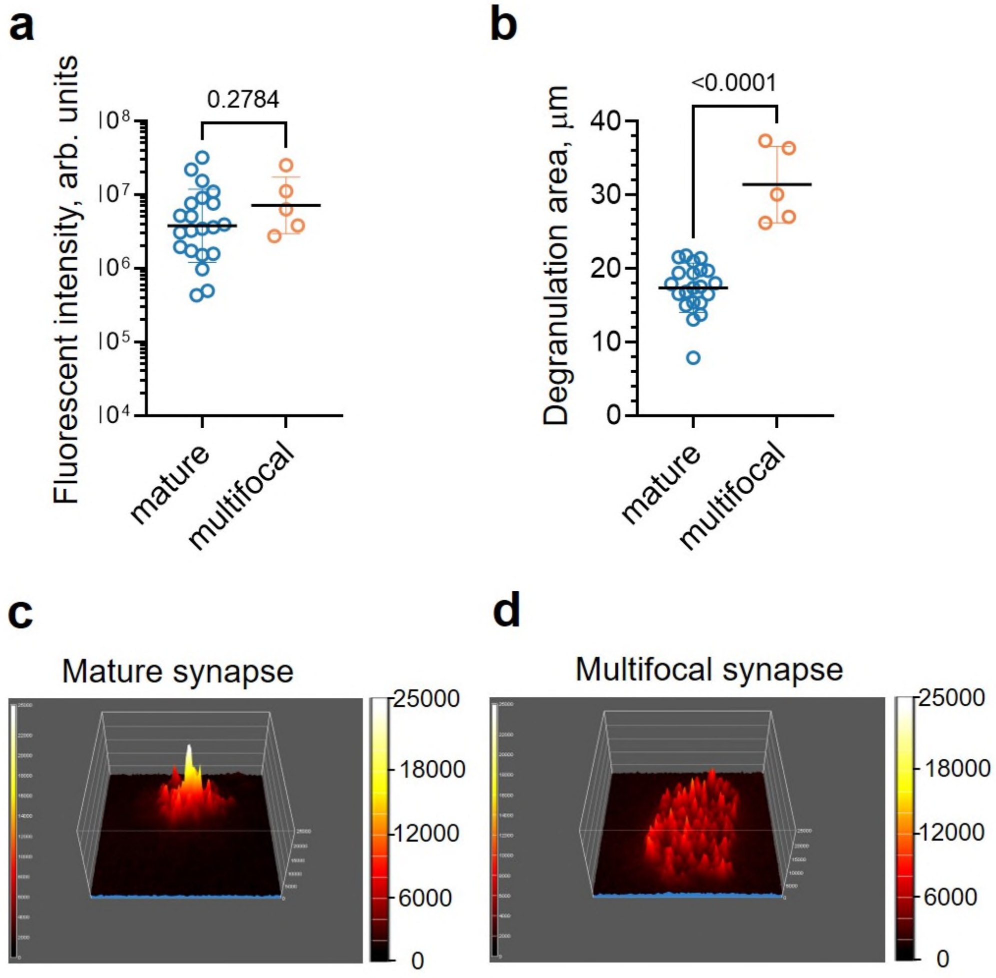

Supplementary Figure 12. CD8 T cells forming mature and multifocal synapses release similar amounts of cytolytic granules; nevertheless, the cells establishing mature synapses show more focused delivery of the granules. EM CD8 T cells from chronic HIV+ donors were exposed to bilayer surface presenting anti-CD3 antibodies and ICAM-1 in the buffer containing fluorescently labeled anti-CD107a Fab fragments. The images of interface were taken by TIRF microscope at 30 minutes after initial T cell-bilayer contact. Regions were drawn around areas where degranulation foci were observed, and areas of those regions were determined. Total fluorescent intensity of the released granules after background subtraction (**a**) and degranulation area (**b**) are presented for cells forming mature (n=21) and multifocal synapses (n=5). Geometric means and geometric SD factors (**a**) and means with SDs (**b**) are shown as horizontal black lines and black error bars. Nonparametric two-tailed Mann–Whitney test (**a**) and two-tailed t test (**b**) were applied to calculate p values indicated on the top of corresponding graphs. One representative experiment is shown (N=3 independent experiments). Source data are provided as a source data file. Representative surface intensity plot of EM CD8 T cell degranulation at mature synapse (**c**) and multifocal synapse (**d**) are shown.
