# Supplementary material for: The immune synapses reveal aberrant functions of CD8 T cells during chronic HIV infection
Source: Nat Commun. 2022 Oct 28;13:6436. doi: 10.1038/s41467-022-34157-0 (PMC9616955; doi:10.1038/s41467-022-34157-0)
Supplement: Supplementary file 3 — Description of Additional Supplementary Files [file 41467_2022_34157_MOESM3_ESM.pdf]

Description of Additional Supplementary Files

Supplementary Movie 1. Formation of mature synapse at interface between CD8 T cell and planar bilayer containing fluorescent-labeled anti-CD3 antibodies and ICAM-1. After the cells were exposed to the bilayer, images were taken every 2 minutes for 30 minutes. The movie was assembled using MetaMorph software (v. 7.7.2.0). Quick segregation of ICAM-1 and anti-CD3 molecules leads to formation of stable pSMAC and cSMAC zones. Movie represents time-dependent appearance of confocal and IRM images at the interface; anti-CD3 antibodies are in green, ICAM-1 molecules are in blue, IRM images are in red.

Supplementary Movie 2. Movie demonstrates time-dependent appearance of bright field images of the same cell as in Supplementary Movie 1. After short period of time, initial unstable contacts of the cell with the bilayer have been stabilized.

Supplementary Movie 3. Formation of TCR/CD3 focal interface between CD8 T cell and planar bilayer containing fluorescent-labeled anti-CD3 antibodies and ICAM-1. The cells were exposed to the bilayer, and images were taken every 2 minutes for 30 minutes. The movie was montaged with MetaMorph software (v. 7.7.2.0). Formation and merger of anti-CD3 antibody clusters that represent adhesion area is demonstrated. Movie represents time-dependent appearance of confocal and IRM images of the entire stack; anti-CD3 antibodies are in green, ICAM-1 molecules are in blue, IRM images are in red.

Supplementary Movie 4. Movie represents time-dependent appearance of bright field images of the same cell as in Supplementary Movie 3. Formation of uropod-like structure on the bilayer surface is observed at anti-CD3 antibodies accumulation spots within a small adhesion area, but the cell body protrudes up and is wobbled around.

Supplementary Movie 5. Formation of kinapse at interface between a CD8 T cell and a planar bilayer containing fluorescent-labeled anti-CD3 antibodies and ICAM-1. The cells were exposed to the bilayer, and images were taken every 2 minutes for 30 minutes. The movie was assembled using MetaMorph software (v. 7.7.2.0). After contacting the bilayer, the cell form transient 'bull-eye' structure which converts because of cell movement into asymmetric kinapse with dSMAC zone under lamellipodium at the leading edge of the cell, middle pSMAC zone, and accumulated anti-CD3 antibodies in the trailing edge. Movie represents the entire stack of confocal and IRM images systematically collected during time of observation; anti-CD3 antibodies are in green, ICAM-1 molecules are in blue, IRM images are in red.

Supplementary Movie 6. Movie represents systematically collected stack of bright field images during observation time of the same cell as in Supplementary Movie 5. The cell movement, formation of leading lamellipodium and trailing uropod are observed.

Supplementary Movie 7. Formation of multifocal synapse at the interface between a CD8 T cell and a planar bilayer containing fluorescent-labelled anti-CD3 antibodies and ICAM-1. After the cells were exposed to the bilayer, images were taken every 2 minutes for 30 minutes. The

movie was montaged with MetaMorph software (v. 7.7.2.0). Clusters of anti-CD3 molecules are dispersed among accumulated ICAM-1. Small clusters fused together to form larger clusters, but complete segregation was not achieved during the period of observation. Movie represents time-dependent collections of the entire stack of confocal and IRM images; anti-CD3 antibodies are in green, ICAM-1 molecules are in blue, IRM images are in red.

Supplementary Movie 8. Movie represents time-dependent appearance of bright field images of the same cell as in Supplementary movie 7. After contact with the bilayer, the cell forms stable lamellipodium structure that increases T cell contact area during immunological synapse formation.

Supplementary Movie 9. Degranulation pattern of CD8 T cell that form mature synapse on bilayer surface containing fluorescent-labeled anti-CD3 antibodies and ICAM-1 in the presence of soluble fluorescent-labeled anti-CD107a Fab fragments. CD8 T cells were allowed to adhere to the bilayer for 2 minutes, and then the images were taken every minute for the entire time of observation. The images were montaged into the movie using Metamorph software (v. 7.7.2.0). TIRF microscopy was utilized for analysis anti-CD3 (green) and anti-CD107a (red) antibodies distribution, and wide-field fluorescent microscopy was exploited to assess ICAM-1 accumulation.

Supplementary Movie 10. Movie represents the same cell as in Supplementary Movie 9. Only released granule and ICAM-1 position are shown. Vast majority of granule were observed inside the ICAM-1 ring, the location where anti-CD3 antibodies accumulated.

Supplementary Movie 11. Degranulation pattern at TCR/CD3 focal synapses that were formed between CD8 T cell and the bilayer presenting fluorescent-labeled anti-CD3 antibodies and ICAM-1 in the presence of soluble fluorescent-labeled anti-CD107a Fab fragments. CD8 T cells were allowed to form contact with the bilayer for 2 minutes, and then the images were taken at a rate of one frame/min. Anti-CD3 (green) and anti-CD107a (red) antibodies position were determined by means of TIRF microscopy, while ICAM-1 (blue) distribution was evaluated with wide-field fluorescent microscopy. The movie was prepared using Metamorph software (v. 7.7.2.0). Pattern of degranulation and position of ICAM-1 and anti-CD3 antibodies are filmed.

Supplementary Movie 12. Movie represents the same cell as in Supplementary Movie 11. Only granule and ICAM-1 position are shown. Degranulation was observed at the large anti-CD3 antibodies spot and fades with time.

Supplementary Movie 13. Degranulation of CD8 T cell that forms kinapse and is moving across bilayer surface. TIRF microscopy utilized for analysis anti-CD3 (green) and anti-CD107a (red) antibodies distribution, and wide-field fluorescent microscopy was applied to assess ICAM-1 (blue) accumulation. CD8 T cells were loaded along with anti-CD107a Fab fragments on the bilayer containing fluorescent-labeled anti-CD3 antibodies and ICAM-1 molecules. After 2 minutes, the images were taken every minute for the entire time of observation and then montaged into the movie using Metamorph software (v. 7.7.2.0). The cell transiently forms

classical bull eye structure with granule inside of ICAM-1 ring. Then the synapse loses the symmetry, and cell started moving to form kinapse structure at the interface. At this stage, the granule release observed mostly in or nearby the area of ICAM-1 accumulation.

Supplementary Movie 14. Movie represents the same cell as in Supplementary Movie 13. Only granule and ICAM-1 position are shown.

Supplementary Movie 15. Degranulation pattern of CD8 T cell that forms multifocal synapse. TIRF microscopy exploited to visualize anti-CD3 (green) and anti-CD107a (red) antibodies distribution, and wide-field fluorescent microscopy was utilized to assess ICAM-1 (blue) accumulation. CD8 T cells were loaded together with anti-CD107a Fab fragments on the bilayer containing fluorescent-labeled anti-CD3 antibodies and ICAM-1 molecules. After 4 minutes, the images were taken every minute for the entire time of observation and then montaged into the movie using Metamorph software (v. 7.7.2.0). The granule release occurs across entire cell-bilayer interface in multiple locations, which are mostly devoid of ICAM-1.

Supplementary Movie 16. Movie represents the same cell as in Supplementary Movie 15. Only granule and ICAM-1 position are shown.
