# Supplementary material for: The immune synapses reveal aberrant functions of CD8 T cells during chronic HIV infection
Source: Nat Commun. 2022 Oct 28;13:6436. doi: 10.1038/s41467-022-34157-0 (PMC9616955; doi:10.1038/s41467-022-34157-0)
Supplement: Supplementary file 20 — Reporting Summary [file 41467_2022_34157_MOESM20_ESM.pdf]

nature portfolio

Corresponding author(s): Yuri Sykulev

Last updated by author(s): 2022/10/09

Reporting Summary

Nature Portfolio wishes to improve the reproducibility of the work that we publish. This form provides structure for consistency and transparency in reporting. For further information on Nature Portfolio policies, see our Editorial Policies and the Editorial Policy Checklist.

Statistics

For all statistical analyses, confirm that the following items are present in the figure legend, table legend, main text, or Methods section.

n/a	Confirmed	
☐	☒	The exact sample size (*n*) for each experimental group/condition, given as a discrete number and unit of measurement
☐	☒	A statement on whether measurements were taken from distinct samples or whether the same sample was measured repeatedly
☐	☒	The statistical test(s) used AND whether they are one- or two-sided<br>*Only common tests should be described solely by name; describe more complex techniques in the Methods section.*
☒	☐	A description of all covariates tested
☐	☒	A description of any assumptions or corrections, such as tests of normality and adjustment for multiple comparisons
☐	☒	A full description of the statistical parameters including central tendency (e.g. means) or other basic estimates (e.g. regression coefficient) AND variation (e.g. standard deviation) or associated estimates of uncertainty (e.g. confidence intervals)
☐	☒	For null hypothesis testing, the test statistic (e.g. F, t, r) with confidence intervals, effect sizes, degrees of freedom and P value noted<br>*Give P values as exact values whenever suitable.*
☒	☐	For Bayesian analysis, information on the choice of priors and Markov chain Monte Carlo settings
☒	☐	For hierarchical and complex designs, identification of the appropriate level for tests and full reporting of outcomes
☒	☐	Estimates of effect sizes (e.g. Cohen's d, Pearson's r), indicating how they were calculated

Our web collection on statistics for biologists contains articles on many of the points above.

Software and code

Policy information about availability of computer code

Data collection	"Flow data were collected with FACSDiva (v.8.01).<br>Confocal images were acquired with Nikon Elements (v. 4.50.00).<br>TIRF images were acquired with MetaMorph (v.7.7.2.0 and 7.10).
Data analysis	Flow Cytometry data were analyzed with FlowJo (v.9.9.4)."<br>Flow Cytometry data were quantified using Bangs Laboratory QuickCal Data Analysis Program (v.2.3).<br>Images were analyzed with Nikon NIS-Elements AR (v. 4.50.00) and MetaMorph (v. 7.7.2.0) software.<br>Statistical analysis and graph generation was conducted with GraphPad Prizm (v.8.0 and 9.0.1).

For manuscripts utilizing custom algorithms or software that are central to the research but not yet described in published literature, software must be made available to editors and reviewers. We strongly encourage code deposition in a community repository (e.g. GitHub). See the Nature Portfolio guidelines for submitting code & software for further information.

Data

Policy information about availability of data

All manuscripts must include a data availability statement. This statement should provide the following information, where applicable:

- Accession codes, unique identifiers, or web links for publicly available datasets
- A description of any restrictions on data availability
- For clinical datasets or third party data, please ensure that the statement adheres to our policy

The raw numbers for Figures 1i, 2a-c, 3b, 4b, 5a and b, 7a and c and Supplementary Figures 1a-c, 3a-c, 5b, 6a-c, 7a-c, 8a-c, 9a-c, 11a and b, 12a and b are available in the Source Data file. Relevant raw microscopy images and flow cytometry data are available from the corresponding author upon reasonable request. Source data are provided with this paper.

Field-specific reporting

Please select the one below that is the best fit for your research. If you are not sure, read the appropriate sections before making your selection.

[x] Life sciences [ ] Behavioural & social sciences [ ] Ecological, evolutionary & environmental sciences

For a reference copy of the document with all sections, see nature.com/documents/nr-reporting-summary-flat.pdf

Life sciences study design

All studies must disclose on these points even when the disclosure is negative.

Sample size	No sample size was calculated for donors tested. Sample size was chosen based on samples availability and the number of cells to be analyzed. Thus, sample size in terms of the number of cells to be analyzed per donor is the most important metric providing an accurate evaluation of the varieties of available cellular types for the analysis. However, there was no preliminary data set available upon which to generate power calculations necessary for the analysis of cell numbers
Data exclusions	Only data affected by technical issues were excluded
Replication	Due to a limited cell availability, only a single sample was tested per individual. Rather than technical replicates performed with the cells from the same individual, samples from several individuals were tested. At least three independent donors were tested per experiment and donor group unless stated otherwise. All reported independent experiments are successful
Randomization	We have three observational study groups, namely, HIV+, HIV+ treated with antiretroviral therapy, and HIV uninfected donors. For each cohort, deidentified and coded samples were randomly chosen from available samples and allocated to the corresponding donor group
Blinding	The sample collection, deidentification and allocation to observational donor groups was performed by personnel that were not involved into the sample analysis.

Reporting for specific materials, systems and methods

We require information from authors about some types of materials, experimental systems and methods used in many studies. Here, indicate whether each material, system or method listed is relevant to your study. If you are not sure if a list item applies to your research, read the appropriate section before selecting a response.

Materials & experimental systems

n/a	Involved in the study	
[ ]	[x]	Antibodies
[ ]	[x]	Eukaryotic cell lines
[x]	[ ]	Palaeontology and archaeology
[x]	[ ]	Animals and other organisms
[ ]	[x]	Human research participants
[x]	[ ]	Clinical data
[x]	[ ]	Dual use research of concern

Methods

n/a	Involved in the study	
[x]	[ ]	ChIP-seq
[ ]	[x]	Flow cytometry
[x]	[ ]	MRI-based neuroimaging

Antibodies

Antibodies used	anti-CD45RO PE CF594 (clone UCHL1): BD Cat# 562299, Lot# 8067791, dilution 1:50<br>anti-CD56 PE Cy7 (clone B159): BD Cat# 560916, Lot# 7319530, dilution 1:100<br>anti-CD45RA BV650 (clone HI100): BD Cat# 563963, Lot# 8129909, dilution 1:166.7

anti-CCR7 APC Cy7 (clone G043H7): Biolegend Cat# 353211, Lot# B314830, dilution 1:50
anti-CD27 BV785 (clone O323): Biolegend Cat# 302827, Lot# B264783, dilution 1:66.7
anti-CD10 BV605 (clone HI10A): Biolegend Cat# 312222, Lot# B217326, dilution 1:100
anti-CD14 BV510 (clone M5E2): Biolegend Cat# 301842, Lot# B251706, dilution 1:200
anti-CD19 PE (clone HIB19): Biolegend Cat# 302208, Lot# B273506, dilution 1:200
anti-CD16 BV510 (clone 3G8): Biolegend Cat# 302048, Lot# B202740 , dilution 1:200
anti-CD4 PE Cy7 (clone RPA-T4): Biolegend Cat# 300512, Lot# B240351, dilution 1:333.3
anti-CD8 V500 (clone SK1): BD Horizon Cat#557696, Lot# 3200606, dilution 1:200
anti-CD27 Alexa 647 (clone O323): Biolegend Cat# 302812, Lot# 235714, dilution 1:100
anti-CD45RO BV650 (clone UCHL1): Biolegend Cat# 304232, Lot# B256838, dilution 1:100
anti-CD3 (clone OKT3): purified from culture supernatant of hybridoma (CRL-8001, ATCC) and labeled with biotin and Alexa Fluor 488, 2 μg/ml
anti-CD107a (clone H4A3): purified from culture supernatant of hybridoma (H4A3, DSHB) and labeled with Alexa Fluor 568, 2 μg/ml
anti-CD54 (anti-ICAM-1, clone YN1.1): purified from culture supernatant of hybridoma (CRL-1878, ATCC) and coupled with CNBr-activated Sepharose 4B
anti-CD11a (anti-LFA-1, clone TS2/4.1.1): purified from culture supernatant of hybridoma (HB-244, ATCC) and labeled with Alexa Fluor 488, 2 μg/ml
anti-CD45RO PE CF594 (clone UCHL1): BD Cat# 562299, Lot# 8067791, dilution 1:50
anti-CD56 PE Cy7 (clone B159): BD Cat# 560916, Lot# 7319530, dilution 1:100
anti-CD45RA BV650 (clone HI100): BD Cat# 563963, Lot# 8129909, dilution 1:166.7
anti-CCR7 APC Cy7 (clone G043H7): Biolegend Cat# 353211, Lot# B314830, dilution 1:50
anti-CD27 BV785 (clone O323): Biolegend Cat# 302827, Lot# B264783, dilution 1:66.7
anti-CD10 BV605 (clone HI10A): Biolegend Cat# 312222, Lot# B217326, dilution 1:100
anti-CD14 BV510 (clone M5E2): Biolegend Cat# 301842, Lot# B251706, dilution 1:200
anti-CD19 PE (clone HIB19): Biolegend Cat# 302208, Lot# B273506, dilution 1:200
anti-CD16 BV510 (clone 3G8): Biolegend Cat# 302048, Lot# B202740 , dilution 1:200
anti-CD4 PE Cy7 (clone RPA-T4): Biolegend Cat# 300512, Lot# B240351, dilution 1:333.3
anti-CD8 V500 (clone SK1): BD Horizon Cat#557696, Lot# 3200606, dilution 1:200
anti-CD27 Alexa 647 (clone O323): Biolegend Cat# 302812, Lot# 235714, dilution 1:100
anti-CD45RO BV650 (clone UCHL1): Biolegend Cat# 304232, Lot# B256838, dilution 1:100
anti-CD3 (clone OKT3): purified from culture supernatant of hybridoma (CRL-8001, ATCC) and labeled with biotin and Alexa Fluor 488, 2 μg/ml
anti-CD107a (clone H4A3): purified from culture supernatant of hybridoma (H4A3, DSHB) and labeled with Alexa Fluor 568, 2 μg/ml
anti-CD54 (anti-ICAM-1, clone YN1.1): purified from culture supernatant of hybridoma (CRL-1878, ATCC) and coupled with CNBr-activated Sepharose 4B
anti-CD11a (anti-LFA-1, clone TS2/4.1.1): purified from culture supernatant of hybridoma (HB-244, ATCC) and labeled with Alexa Fluor 488, 2 μg/ml

Validation

1. Biolegend:
Anti-CCR7 APC Cy7 (clone G043H7, cat# 353211); validation: staining of human peripheral blood lymphocytes
Anti-CD27 BV785 (clone O323, cat# 302827); validation: staining of human peripheral blood lymphocytes
Anti-CD10 BV605 (clone HI10A; cat# 312222); validation: staining of human peripheral blood granulocytes
Anti-CD14 BV510 (clone M5E2, cat# 301842); validation: staining of human peripheral blood monocytes
Anti-CD19 PE (clone HIB19, cat# 302208); validation: staining of human peripheral blood lymphocytes
Anti-CD16 BV510 (clone 3G8, cat# 302048); validation: staining of human peripheral blood lymphocytes
Anti-CD4 PE Cy7 (clone RPA-T4, cat# 300512); validation: staining of human peripheral blood lymphocytes
Anti-CD27 Alexa 647 (clone O323, cat# 302812); validation: staining of human peripheral blood lymphocytes
Anti-CD45RO BV650 (clone UCHL1, cat# 304232); validation: staining of human peripheral blood lymphocytes

2. BD:
Anti-CD45RO PE CF594 (clone UCHL1, cat# 562299); validation: expression on human peripheral blood lymphocytes
Anti-CD56 PE Cy7 (clone B159, cat# 560916); validation: expression on human peripheral blood lymphocytes
Anti-CD45RA BV650 (clone HI100, cat# 563963); validation: expression on human peripheral blood lymphocytes
Anti-CD8 V500 (clone SK1, cat#557696); validation: expression on human peripheral blood lymphocytes

3. In-house purified antibodies:
Anti-CD3 biotin/Alexa 488 (clone OKT3), purified from culture supernatant of hybridoma CRL-8001, ATCC) and labeled with biotin and Alexa Fluor 488. The antibody has been previously validated in literature (Chang T.W. et al. Proc Natl Acad Sci U S A. (1981) 78:1805–8) and in our previous work using glass-supported lipid bilayers (Steblyanko M. et al. J Vis Exp. (2018) 137:58143). In current study, the antibody utilized for preparation of the bilayers.
Anti-CD54 (anti-ICAM-1, clone YN1/1), purified from culture supernatant of hybridoma CRL-1878, ATCC) and conjugated with Sepharose 4B. The antibody has been previously validated in literature (Horley K.J. et al. EMBO J. (1989) 8(10): 2889–2896). In our previous published work using glass-supported lipid bilayers, the antibody utilized for purification of recombinant ICAM-1 (Steblyanko M. et al. J Vis Exp. (2018) 137:58143). In current study, the antibody utilized for purification of recombinant ICAM-1.
Anti-CD11a Alexa 488 (anti-LFA-1, clone TS2/4.1.1), purified from culture supernatant of hybridoma HB-244, ATCC) and labeled with Alexa Fluor 488. The antibody has been previously validated in literature (Huang C. and Springer T.A. Proc. Natl. Acad. Sci. U S A (1997) 94(7):3162-7) and in our previous work as a reagent for attachment of T cells to polylysine coated glass surface (Anikeeva N. Nature Communications (2016) v. 7: 13264). In current study, the antibody utilized for staining of purified human T cells.
Anti-CD107a Alexa 568 (clone H4A3), purified from culture supernatant of hybridoma H4A3, DSHB and labeled with Alexa Fluor 568.

The antibody has been previously validated in literature (Chen J.W. et al. The Journal of Cell Biology (1985) v. 101: 85-95) and in our previous work to study T cell degranulation using functional assay and glass-supported lipid bilayers (Betts M.R. et al. Journal of Immunological Methods (2003) v. 281: 65-78; Steblyanko M. et al. J Vis Exp. (2018) 137:58143). In current study, the antibody utilized for monitoring T cell degranulation on glass-supported lipid bilayers.

Eukaryotic cell lines

Policy information about cell lines

Cell line source(s)	Hybridoma OKT3 producing antibodies recognizing human CD3zeta and hybridoma TS2/4.1.1 secreting antibodies against human LFA-1 were purchased from ATCC. Hybridoma H4A3 secreting antibody against CD107a (LAMP-1) was kindly provided by Dr. J. Thomas August, Department of Pharmacology and Molecular Sciences, Johns Hopkins Medical School. Hybridoma YN1.1 (ATCC) producing antibodies against ICAM-1 was kind gift from Michael Dustin, Skirball Institute of Biomolecular Medicine, New York University.
Authentication	All antibody producing hybridoma cell lines were not authenticated. The antibody produced by the hybridoma cell lines were purified and validated.
Mycoplasma contamination	The hybridoma cell lines were mycoplasma negative.
Commonly misidentified lines (See ICLAC register)	None of hybridoma cell lines used in the study are listed in the ICLAC register.

Human research participants

Policy information about studies involving human research participants

Population characteristics	Samples obtained from deidentified HIV- and HIV+ individuals over the age of 18 were used for these studies. Relevant information adhering to BRISQ Tier 1 requirements provided in the manuscript.
Recruitment	All samples used in these studies were derived from IRB approved cohorts of patients at the University of Pennsylvania, and the University of Toronto. HIV+ and HIV- donors were recruited into these cohorts based on parameters established in their respective studies at each institution. No selection biases have been identified.
Ethics oversight	All enrolled participants provided written informed consent conform to Helsinki Declaration, and the protocol used was approved by the Institutional Review Board of the University of Pennsylvania (IRB 809316) and the University of Toronto (REB 12-378). HIV+ donors did not receive compensation beyond minimal costs associated with travel and time on the donation day. Healthy HIV-negative PBMC samples were obtained from the University of Pennsylvania Human Immunology Core. HIV- donors received $175 for apheresis donation to the University of Pennsylvania Center for AIDS Research Human Immunology Core. All data specimens were coded to protect confidentiality.

Note that full information on the approval of the study protocol must also be provided in the manuscript.

Flow Cytometry

Plots

Confirm that:

- [x] The axis labels state the marker and fluorochrome used (e.g. CD4-FITC).
- [x] The axis scales are clearly visible. Include numbers along axes only for bottom left plot of group (a 'group' is an analysis of identical markers).
- [x] All plots are contour plots with outliers or pseudocolor plots.
- [x] A numerical value for number of cells or percentage (with statistics) is provided.

Methodology

Sample preparation	Cryopreserved PBMC were thawed, rested overnight, and sorted. Full details are provided in the Methods section of the manuscript. Analysis of LFA-1 expression: the analyzed CD8 T cell subsets are purified by magnetic sorting as described in the manufactiurer's protocol. The cells were live stained for 30 minutes at 4oC, washed, fixed, resuspended in FACS buffer and analyzed by flow cytometry.
Instrument	Facs Aria, BDLSRFortressa, BD LSRII cytometers
Software	Becton Dickinson Diva software, FlowJo (v. 9.9.4)
Cell population abundance	The recovery of the different populations varied based on the memory subset sorted. All sorted populations were 99% pure or higher.
Gating strategy	Populations of naive T cells were sorted based on the gating strategy shown in Supplementary Information. Analysis of LFA-1

expression: Cells are gated on the live cell population by forward and side scatter and then on single cells by height vs area scatter. Positive population is determined using negative control. Example of high and low LFA-1 expressing cells gating is provided in Supplementary Information. The same gates are applied to analyze different T cell subsets. For each experiment, fluorescent calibrating beads are used to quantity numbers of fluorophores per cells

☒ Tick this box to confirm that a figure exemplifying the gating strategy is provided in the Supplementary Information.
